# Identification of a Serine Proteinase Homolog (Sp-SPH) Involved in Immune Defense in the Mud Crab *Scylla paramamosain*

**DOI:** 10.1371/journal.pone.0063787

**Published:** 2013-05-28

**Authors:** Qiu-xia Zhang, Hai-peng Liu, Rong-yuan Chen, Kai-li Shen, Ke-jian Wang

**Affiliations:** State Key Laboratory of Marine Environmental Science, College of Ocean & Earth Science, Xiamen University, Xiamen, China; Federal University of Rio de Janeiro, Brazil

## Abstract

Clip domain serine proteinase homologs are involved in many biological processes including immune response. To identify the immune function of a serine proteinase homolog (Sp-SPH), originally isolated from hemocytes of the mud crab, *Scylla paramamosain*, the Sp-SPH was expressed recombinantly and purified for further studies. It was found that the Sp-SPH protein could bind to a number of bacteria (including *Aeromonas hydrophila, Escherichia coli, Staphylococcus aureus*, *Vibrio fluvialis, Vibrio harveyi* and *Vibrio parahemolyticus*), bacterial cell wall components such as lipopolysaccharide or peptidoglycan (PGN), and β-1, 3-glucan of fungus. But no direct antibacterial activity of Sp-SPH protein was shown by using minimum inhibitory concentration or minimum bactericidal concentration assays. Nevertheless, the Sp-SPH protein was found to significantly enhance the crab hemocyte adhesion activity (paired t-test, *P*<0.05), and increase phenoloxidase activity if triggered by PGN in vitro (paired t-test, *P*<0.05). Importantly, the Sp-SPH protein was demonstrated to promote the survival rate of the animals after challenge with *A. hydrophila* or *V. parahemolyticus* which were both recognized by Sp-SPH protein, if pre-incubated with Sp-SPH protein, respectively. Whereas, the crabs died much faster when challenged with *Vibrio alginolyiicus*, a pathogenic bacterium not recognized by Sp-SPH protein, compared to those of crabs challenged with *A. hydrophila* or *V. parahemolyticus* when pre-coated with Sp-SPH protein. Taken together, these data suggested that Sp-SPH molecule might play an important role in immune defense against bacterial infection in the mud crab *S. paramamosain*.

## Introduction

Invertebrates rely solely on innate immunity against invading pathogens. These immune responses are triggered by the recognition and binding of pattern recognition proteins (PRPs) to the surface molecules such as lipopolysaccharide (LPS) and peptidoglycan (PGN) of bacterial cell walls, and β-1, 3-glucan of fungal cell walls, of the invading microorganism [1]. Current studies have identified many PRRs (LPS-, β-1, 3-glucan-, peptidoglycan-binding proteins, lectins and hemolins) from a variety of invertebrates and their different biological functions like activation of Toll/IMD pathway [2], [3], [4] and prophenoloxidase (proPO) system [5], [6], [7]. Recently, clip domain serine proteinases and clip domain serine proteinase homologs (clip-SPHs) have been shown to participate in various biological functions including immunity [8], [9]. In arthropods, the clip-SPHs are involved in antimicrobial defense in the horseshoe crab *Tachypleus tridentatus* [10], as an immune molecule in the mosquito *Anopheles gambiae* [11], in the activation/regulation of the proPO-system in insects such as *Anopheles* [12], *Tenebrio molitor* [13] and *Manduca sexta* [14], in pattern recognition, opsonization and cell adhesion activity in freshwater crayfish *Pacifastacus leniusculus* [15], [16]. Therefore, the studies of proteinases or proteinase homologs with clip domains are essential for elucidating the innate immune responses against invading pathogens in invertebrates.

In invertebrates, hemocytes contain a large number of immune factors which are critical for pathogen sensing, immune signal transduction and microbial killing effects. Previously, we have isolated a SPH protein (Sp-SPH) from hemocyte lysate supernatant (HLS) of a crustacean, the mud crab *Scylla paramamosain*, via a live bacterial-affinity matrix and determined the full-length cDNA sequence of Sp-SPH gene as well as its expression profile post bacterial infection [17]. To further explore the immune functions of the Sp-SPH molecule, recombinant Sp-SPH protein was expressed in the yeast *Pichia pastoris*. The purified recombinant protein was then characterized for immune roles such as bacterial recognition, binding activity to bacterial or fungal associated components, hemocyte adhesion activity, proPO activation and immune protection against bacterial challenge in the mud crab *S. paramamosain*.

## Materials and Methods

### Preparation of recombinant Sp-SPH protein

To further characterize the Sp-SPH in terms of biological activity, the recombinant Sp-SPH was expressed in the yeast *P. pastoris*. The forward primer introduced an EcoR I site (underlined) as 5′TTTGAATTCGGACCAAGGGAGCGGCGCC-3′. The reverse primer was designed as 5′-AAGCGGCCGCTCA**ATGATGATGATGATGG TG**ATCGTAGCCCCAGTAGTCC-3′ with an endonuclease site Not I (underlined) and a 6× His-tag (bold) at the carboxyl terminus of Sp-SPH gene. These two primers were used to amplify the ORF of Sp-SPH by PCR using mud crab hemocyte cDNA. The cDNA was prepared as described previously [17]. PCR reaction was prepared as follows: 94°C for 45 s, 60°C for 30 s and 72°C for 90 s with 30 cycles. The PCR product was ligated into vector pPIC9K (Invitrogen) and the ligation mixture was transformed into *E. coli* DH5α. The constructed recombinant plasmid pPIC9K-Sp-SPH was confirmed by DNA sequencing. The recombinant plasmid of pPIC9K-Sp-SPH was then linearized with *Sac* I and transformed into competent *P. pastoris* GS115 cells by electroporation using the Bio-Rad gene pulser Xcell™. And the pPIC9K vector was also linearized and transformed into *P. pastoris* GS115 cells as a negative control. These transformants were selected on MD plates and incubated at 30°C for 2–3 days. Positive clones were next screened by PCR with primers 5′AOX (GACTGGTTCCA ATTGACAAGC) and 3′AOX (GCAAATGGCATTCTGACATCC) before subjected to recombinant expression induced by 0.5% methonal. The clones of each recombinant expressing relatively high amount of recombinant protein were selected for large-scale production. After induction with 0.5% of methonal for 24 h, the protein containing supernatant was separated from the yeast pellet and dialyzed against 50 mM sodium phosphate buffer (50 mM PBS, 50 mM NaCl, pH 8.0) at 4°C before purified by immobilized metal affinity chromatography. After 24–36 h dialysis, the supernatant containing the secreted component of Sp-SPH protein was collected by centrifugation at 15,000 g for 40 min at 4°C. The collected supernatant was filtered with a 0.45 μm filter membrane and then loaded on a HisTrap FF crude column (GE Healthcare Life Sciences) equilibrated with binding buffer (20 mM PBS, 50 mM NaCl, and 10 Mm imidazole, pH 8.0). The column was washed with binding buffer and then eluted with a gradient of imidazole formed by binding buffer and elution buffer (20 mM PBS, 500 mM NaCl, and 1 M imidazole, pH 8.0). The eluted fractions were collected and dialyzed twice against 20 mM sodium phosphate buffer (20 mM PBS, 20 mM NaCl, pH 8.0), and finally dialyzed in Milli-Q water for 36 h at 4°C. The purified recombinant Sp-SPH was analyzed by 12% SDS–PAGE combined with Coomassie Brilliant Blue staining and the concentration was determined by Bradford method as previously described [6]. The recombinant Sp-SPH was also verified by MALDI-TOF/TOF mass spectrometry. The recombinant protein with a purity of more than 90% was frozen and stored at −80°C for later use.

### Determination of Sp-SPH protein binding activity to bacteria or microbial associated molecule

The recombinant Sp-SPH protein was investigated for binding to different bacteria using a method described by Lee and Söderhäll [15]. Shortly, the Gram-negative bacteria (*Aeromonas hydrophila, Aeromonas sobria, Escherichia coli, Pseudomonas stutzeri, Vibrio alginolyiicus, Vibrio fluvialis, Vibrio harveyi, Vibrio parahemolyticus)* and the Gram-positive bacteria (*Staphylococcus aureus)* were used for testing the specific binding property of the Sp-SPH protein. The cultured bacteria in mid-logarithmic growth phase (OD_600_ of 0.6) were fixed in 3.7% (w/v) formaldehyde by gently shaking at 37°C for 1 h to terminate the enzymatic activity of bacteria. Then the fixed cells were harvested by centrifugation with 2,000 g for 10 min at 4°C followed by washing twice with 1× PBS, and then resuspended in 1× PBS. The purified protein (25 µg of the Sp-SPH protein) was incubated with 0.5 ml of bacterial suspension containing 3.0×10^8^ cells with gentle shaking at 4°C for 30 min, and then centrifuged with 2,000 g at 4°C for 10 min. After that, the supernatant containing unbound protein was removed and the pellet was resuspended and washed for five more times with 1× PBS buffer. Bound proteins were finally eluted from the bacteria by 0.1 M citric acid, pH 2.0. The supernatants containing the eluted bound proteins were transferred into new tubes and concentrated by adding 1/10 volume of trichloroacetic acid and kept on ice for 30 min followed by centrifugation at 15,000 g for 15 min. The resulting protein pellets were resuspended directly in 1× SDS-PAGE loading buffer. Bacteria treated with PBS buffer only were used as control. The eluted fractions (bound protein) were analyzed on 12% (w/v) SDS-PAGE, then transferred to nitrocellulose and subjected to immunoblotting using the anti-His antibody (1:1000, Novagen).

### Binding property of the purified Sp-SPH protein to bacterial associated components

LPS (purified by phenol extraction from *Escherichia coli* 055:B5), PGN (insoluble Lysine -type peptidoglycan and soluble polymeric Lysine-type PGN from *Staphylococcus aureus* cell wall component) and β-1,3-glucan were all purchased from Sigma company and they were tested by ELISA using the method as previously described by Gonzalez et al. [18]. Briefly, the bacterial components (LPS, PGN or β-1,3-glucan solution) were prepared in 100 mM Na_2_CO_3_, 20 mM EDTA, pH9.6 and 50 µL/well contained 3 µg of LPS, PGN or β-1,3-glucan) coated on the bottom of the 96-well ELISA plate and the content was dried up completely for 2 h at 60°C. Then, the redundant LPS, PGN or β-1,3-glucan were washed away by PBS buffer and the non-specific binding were blocked with 5% (w/v) BSA in PBS buffer for 1 h at room temperature. Binding of the serial dilution of the purified Sp-SPH protein (0∼200 µg/mL, 100 µL/well) was carried out for 1 h at room temperature. The wells without recombinant protein incubation were used as control treatments. After washing the excess protein with PBS buffer containing 0.05% Tween 20, the ELISA plate was hybridized with the mouse anti-SPH antibody (1:1000 dilution, 50 µL/well) for 2 h at 37°C. The samples were washed three times as above and followed by incubation with HRP-labeled Goat Anti-Mouse IgG (1:1,000 dilution, 50 µL/well) at 37°C for 1 h. The colorimetric reaction was next detected by adding 100 µL of TMB. After the sufficient blue color formed, 1 M H_2_SO_4_ was added to terminate the reaction. Absorbance of each well was measured at 450 nm by a Multifunctional microplate reader (GENios). The results from three experiments were used for statistical analysis. The binding results were analyzed by Scatchard plot analysis. The binding parameters, apparent dissociation constant Kd, and the maximum binding (Amax), were determined by non-linearly fitting as A  =  Amax [L]/(Kd + [L]), where A was the absorbance at 450 nm and [L] was the protein concentration.

### Examination of mud crab hemocyte adhesion activity mediated by Sp-SPH protein

The cell adhesion assay was performed according to Current Protocols in Cell Biology Online [19] and was measured as previously described [20], 21. Reactions were carried out at 22°C if no particular instruction was stated. Briefly, 100 µL of several concentrations of recombinant Sp-SPH protein (0, 19.2, 38.4, 48, or 96 µg/mL) was added into each well of an ELISA plate followed by incubation overnight at 4°C. Then 100 µL blocking solution (5% BSA in PBS) was added to each well and kept for 1 h. Live healthy inter-molting male crabs, *S. paramamosain* (200±50 g), were bought from a local commercial crab farm in Zhangpu, Fujian, China. The mud crab hemocyte was prepared as previously described [22]. Briefly, the haemolymph was collected and mixed equally with crab anticoagulant solution (NaCl 510 mM; glucose 100 mM; citric acid 200 mM; Na-citrate 30 mM; EDTA-Na 210 mM; pH 7.3) [23] on the ice followed by centrifugation at 800×g for 5 min at 4°C. The resulting hemocyte pellets were washed once with anticoagulant solution, then suspended in modified L-15 medium (L-15, additional NaCl 5 g/L, and glucose 1 g/L). One hundred microliter of hemocyte suspension (1×10^6^ cells/m L) was added into each well and incubated for 20 min. The hemocyte viability was examined by the fluorochrome propidium iodide (PI) (Sigma) [24], [25]. Briefly, PI was added to the hemocyte with a final concentration of 1 μg/mL and the cells were then incubated for another 15 min at room temperature. PI stained hemocytes were detected using an inverted fluorescence microscope (Axio vert 200). For cell adhesion activity test, the hemocytes were washed and fixed in 5% glutaraldehyde for 20 min followed by washing and staining with crystal violet solution (0.1% in 200 mM 2-(N -morpholino) ethanesulfonic acid, pH 6.0) for 1 h. After washing with distilled water for three times, each well was added with 100 µL of acetic acid (10%, v/v) to dissolve the crystal violet. After incubation for 5 min, the percentage of attached cells was assessed by measuring the OD_595nm_ with Multifunctional microplate reader (GENios).

### Phenoloxidase activity affected by Sp-SPH protein in mud crab HLS

To test whether Sp-SPH could induce the proPO activation in crabs when they were subjected to pathogenic bacteria, we determined PO activity in crab HLS after addition of recombinant Sp-SPH protein. The crab haemolymph samples were prepared as previously described [22]. Briefly, the haemolymph was prepared as described above. The resulting hemocyte pellets were washed once with anticoagulant solution, then suspended in homogenization buffer (10 mM sodium cacodylate containing 5 mM CaCl_2_, pH 7.0) and sonicated on the ice. After sonication, the mixture was subjected to centrifugation at 15,000×g for 10 min at 4°C. The supernatant was then collected and used as HLS for further experiments. The protein concentrations were determined by Bradford method as previously described [26]. The proPO activation assay was performed by mixing 20 µL of freshly prepared HLS (containing 60 µg of total protein), 10 µL of Sp-SPH (0.25 µg) (a mud crab antilipopolysaccharide factor 2-Sp-ALF2 and BSA were empolyed as the protein controls), 10 µL of PGN (1 µg), and 25 µL of L-Dopa (3 mg/ml) in a 96-well plate at 20°C. Double distilled water was added to get the total reaction volume of 100 µL. The wells supplied with HLS, HLS+ PGN or HLS+Sp-SPH were used as the controls. The absorbance was measured at 490 nm within 30 min. One Unit of PO activity was defined as an absorbance change of 0.001 at A_490_ per mg protein/min.

### Cumulative mortality of mud crab challenged with bacteria pre-coated with Sp-SPH protein

To further characterize the Sp-SPH in terms of immune protection against invading bacteria, we investigated the mortality of mud crabs (from the same crab farm as described above) after challenge of the selected pathogenic bacteria, *A. hydrophila* or *V. parahemolyticus*, which could be recognized by Sp-SPH protein. Another bacterium not recognized by Sp-SPH protein, *V. alginolyiicus*, was also tested to compare to those of *A. hydrophila* and *V. parahemolyticus*. Briefly, the cultured *A. hydrophila*, *V. parahemolyticus*, or *V. alginolyiicus* in mid-logarithmic growth phase was harvested by centrifugation with 2,000 g for 10 min at 4°C followed by washing twice with 1×PBS, and then resuspended in 1×PBS. The purified protein (25 µg of the Sp-SPH protein) was incubated with 0.5 ml of *A. hydrophila*, *V. parahemolyticus* or *V. alginolyiicus* suspension containing 3.0×10^8^ cells with gentle shaking at 4°C for 30 min. *A. hydrophila*, *V. parahemolyticus* or *V. alginolyiicus* treated with PBS buffer only was used as a control, respectively. After incubation, the mixtures were then centrifuged with 2,000 g at 4°C for 10 min. The pellet was resuspended and washed for five more times with 1×PBS buffer. Four hundred microliter of 1.3×10^7^ cells coated with the Sp-SPH protein or treated with PBS (as a control) were injected into mud crab via the second leg of mud crab. Ten animals were used for each group and they were kept in sea water at 28°C. The mortality was recorded hourly. This experiment was repeated three times.

### Statistical analyses

All statistical analyses were carried out by using SPSS statistics software (SPSS Inc, Chicago, Illinois). The significant difference was defined as *P*<0.05.

## Results and Discussion

### Preparation of recombinant Sp-SPH protein

**Figure 1:**
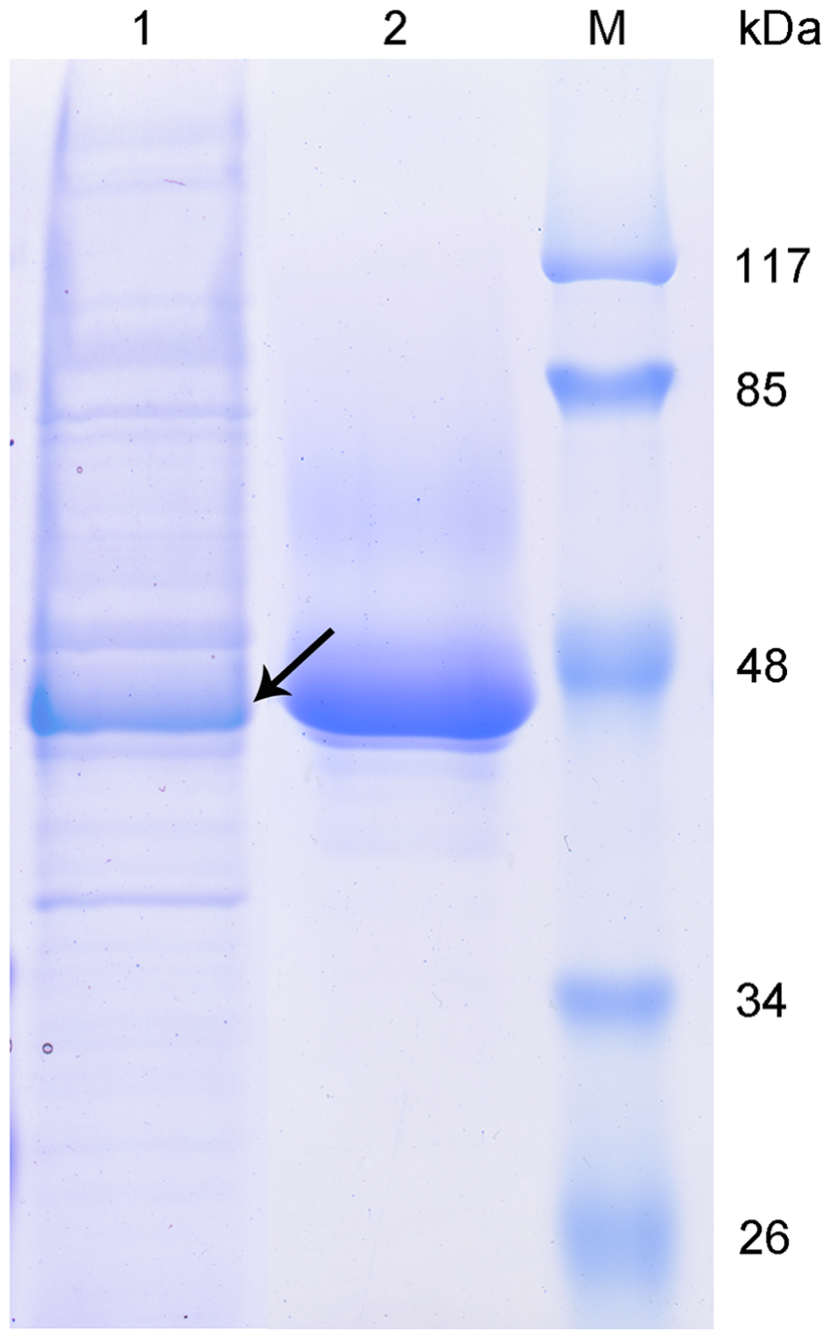
SDS–PAGE analysis of the purified Sp-SPH protein. The recombinant Sp-SPH protein was purified by using Ni2+ affinity chromatography via a 6× His tag as described above. Lane M, molecular weight marker; lane 1, purified Sp-SPH protein; lane 2, cultured medium from pPIC9K/Sp-SPH recombinant clone induced by methonal before protein purification.

To further explore the immune functions of the Sp-SPH molecule involved in crab immune responses, we expressed recombinant Sp-SPH protein in the yeast *P. pastoris* and purified the recombinant protein. As indicated with an arrow in Fig. 1, the recombinant protein showed a major band about 46 kDa as the Sp-SPH molecule, which was in agreement with the calculated molecular mass based on their deduced amino acid sequences including six histidine residues. The recombinant Sp-SPH protein was also verified by MALDI-TOF/TOF mass spectrometry analysis in which several peptide fragments corresponding to the deduced protein sequences of Sp-SPH were confirmed (data not shown).

### Determination of Sp-SPH protein binding activity to bacteria or microbial associated molecules

**Figure 2:**
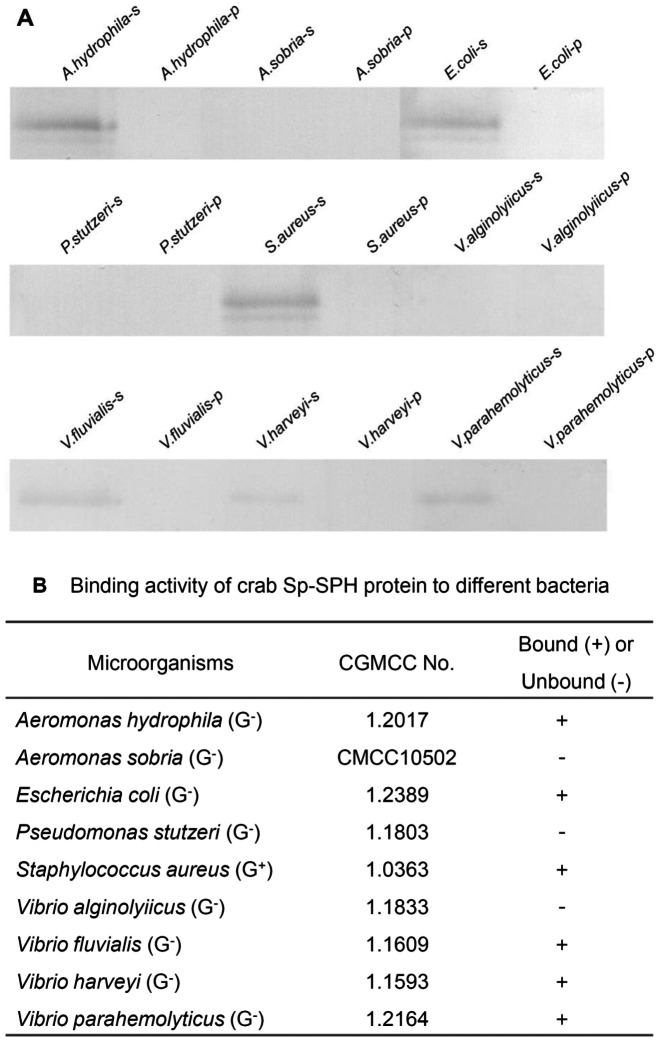
Binding activity of recombinant Sp-SPH protein to different bacteria. The recombinant Sp-SPH protein was incubated with formaldehyde-fixed bacteria. After incubation, the supernatants were separated by centrifugation. The pellets were then washed with PBS and the bound proteins were eluted with SDS-PAGE loading buffer followed by electrophoresis. (A) All eluted samples were examined by Western blot analysis under reducing condition with the employment of the anti-His antibody. –s: bacterium + Sp-SPH protein; -p: bacterium + PBS. (B) Summery of binding affinity of Sp-SPH protein to the bacteria selected.

**Figure 3:**
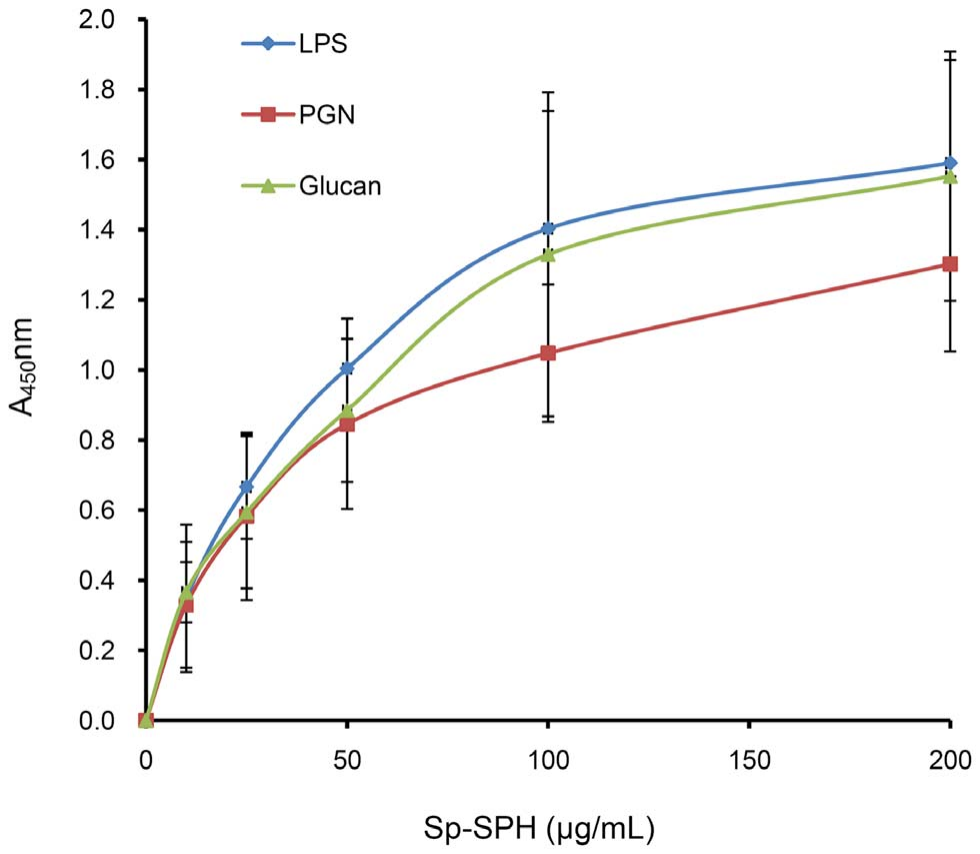
Analysis of the binding affinity between recombinant Sp-SPH protein and the bacterial or fungal associated molecules. The binding affinity of Sp-SPH protein to LPS, PGN or β-1, 3-glucan was tested by ELISA. Absorbance of each well was measured at 450 nm with a Multifunctional microplate reader (GENios). The binding parameters, apparent dissociation constant Kd, and the maximum binding (Amax), were determined by non-linearly fitting as A  =  Amax [L]/(Kd+ [L]). Diamond:lipopolysaccharide (LPS); Square: peptidoglycan (PGN); Triangle: β-1, 3-glucan. The data were representative of the average value of four repeated experiments. Bars indicated mean ± S.E. (*n* = 4).

With the recombinant protein prepared, the binding ability of the Sp-SPH protein to bacteria was investigated. By incubation with the bacteria as described above, the bound recombinant Sp-SPH protein was assessed by Western blotting with the employment of the anti-His antibody. As indicated in Fig. 2, the Sp-SPH protein exhibited higher binding affinity to two Gram-negative bacteria including *A. hydrophila* and *E. coli*, and one Gram-positive bacteria *S. aureus*. Lower binding affinity was observed for *V. fluvialis*, *V. harveyi* and *V. parahemolyticus* if compared to those of bacteria described above. However, no binding activity could be found for *A. sobria*, *P. stutzeri* and *V. alginolyiicus* under the same experimental condition. This result suggested that the Sp-SPH protein could recognize different bacteria, which might lead to activation of immune signaling pathway after its recognition, indicating that this Sp-SPH protein may play a role in immune defense in the mud crab. Besides, the binding characters of Sp-SPH protein to different microbial associated molecules were also examined by using the ELISA, and it was found that the recombinant Sp-SPH protein was bound to LPS, PGN and β-1, 3-glucan in a concentration dependent manner, in which Sp-SPH clearly exhibited binding affinity to LPS, PGN and β-1, 3-glucan at as low as 10 µg/mL. Additionally, the binding activity of Sp-SPH protein towards LPS and β-1, 3-glucan was slightly stronger than that of PGN (Fig. 3). By means of the Scatchard plot analysis, the apparent dissociation constant (Kd) for the binding of the recombinant Sp-SPH protein to LPS, β-1,3-glucan and PGN was 3.2×10^−5^M, 3.1×10^−5^M and 2.1×10^−5^M, respectively. These results suggested that Sp-SPH protein functioned as an immune recognition factor by its binding to the cell walls or constituent carbohydrate such as LPS or PGN for bacteria and β-1, 3-glucan for fungi. On the other hand, Sp-SPH protein could not bind to all the bacteria tested. Based on the results above, it can be concluded that the bacterial recognition mediated by Sp-SPH protein might be bacteria specific due to various molecular structures of the bacterial cell wall components present on their surfaces. Further studies for this case will be helpful for the interpretation of the bacterial-specific defense mediated by Sp-SPH protein in the mud crab. However, unlike certain SPH-containing proteins clearly showing antimicrobial activity in horseshoe crab [10] or human [27], no direct antimicrobial activity with Sp-SPH protein was observed by minimum inhibitory concentration or minimum bactericidal concentration assays (data not shown). Taken together, these data indicated that Sp-SPH protein may function as an innate immune recognition molecule, but not a direct bactericidal factor, in the host defense of the mud crab.

### Examination of mud crab hemocyte adhesion activity mediated by Sp-SPH protein

**Figure 4:**
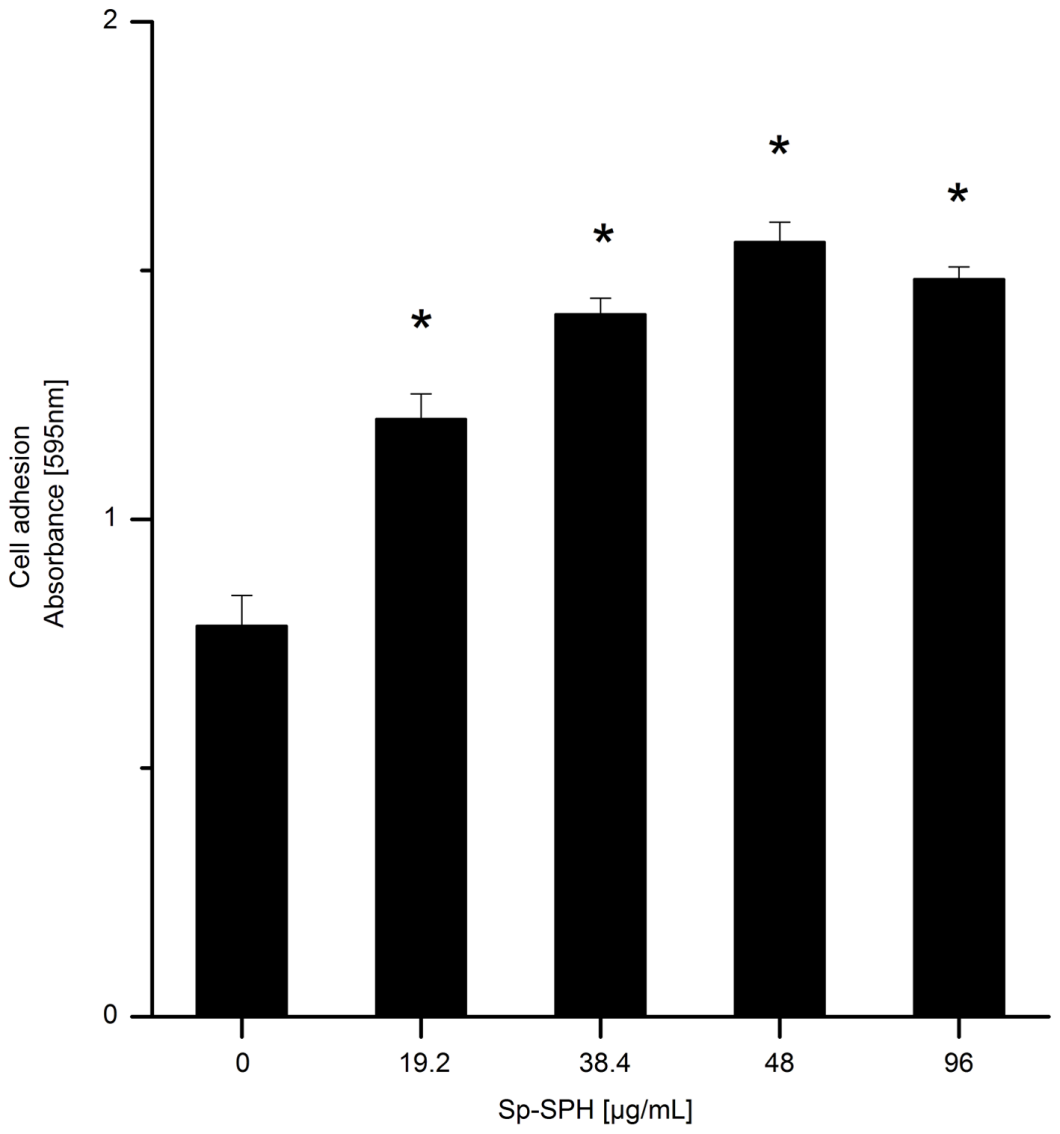
Determination of hemocyte adhesion activity of the mud crab mediated by Sp-SPH recombinant protein. Different concentrations of Sp-SPH recombinant protein were used for coating the ELISA plate followed by addition of crab hemocyte suspension. After washing, the cell adhesion was assessed by measuring the OD_595nm_ value with Multifunctional microplate reader (GENios). *: Significant differences in hemocyte adhesion of Sp-SPH protein treated samples compared to that of non-Sp-SPH protein control (paired *t*-test, *P*<0.05). This experiment was repeated for four times. The results were shown as means ± standards errors (*n* = 4).

It is known that cell adhesion is imperative in the immune system of invertebrates, since it is the initial stage in many cellular responses in innate immunity such as hemocyte spreading, nodule formation, encapsulation, hemocyte aggregation and phagocytosis. Many cell adhesion molecules like clip-domain SPHs are involved in invertebrate immunity [28]. To investigate whether mud crab Sp-SPH protein, as a kind of clip-domain containing molecule, could function as a cell adhesion molecule, the recombinant Sp-SPH protein was tested for its effect on crab hemocyte adhesion in vitro. To exclude the effect of Sp-SPH on cell viability, we firstly determined the hemocyte viability after its preparation for the cell adhesive assay using PI for cell staining (see Figure S1). Both Sp-SPH protein treated crab hemocytes and control hemocytes showed similar cell survival rate (about 96%, see Figure S2). This result obviously indicated that Sp-SPH protein did not affect on the crab hemocyte viability. As shown in Fig. 4 and Figure S3, the recombinant Sp-SPH protein exhibited a significantly higher hemocyte adhesion activity compared to the non-Sp-SPH containing samples. The highest hemocyte adhesion activity was observed with the Sp-SPH concentration of 48 µg/mL tested, which clearly demonstrated that the Sp-SPH protein could act as a cell adhesive molecule in the mud crab. In contrast to the SP-like domain alone in freshwater crayfish, the whole mas-like protein can serve as an opsonin to enhance bacterial clearance [15], [16]. To determine whether Sp-SPH protein had opsonic property, since it had similar SPH structure to the mas-like protein mentioned above, the hemocyte phagocytosis assay towards FITC-labeled *V. parahemolyticus* pre-coated with Sp-SPH protein was carried out in mud crab. But no significant opsonic activity of this Sp-SPH was observed under our experimental condition (data not shown). Similar finding has been reported with a shrimp c-SPH protein, which also showed cell adhesion activity but without opsonic activity as well as direct antimicrobial activity [19]. These data together indicated that clip domain SPH containing molecules could promote the host cellular immune responses by increasing cell adhesion activity in crustaceans.

### Phenoloxidase activity enhanced by Sp-SPH protein in the HLS of mud crab

**Figure 5:**
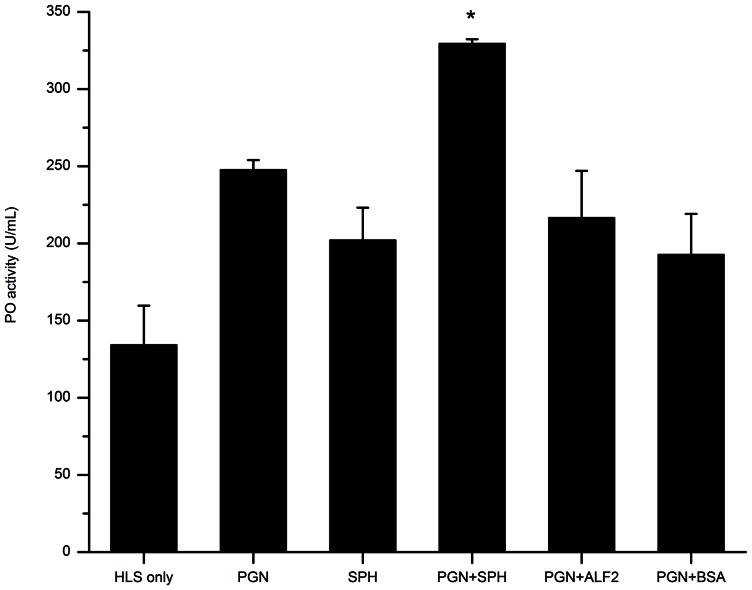
Phenoloxidase activity enhanced by Sp-SPH protein in the HLS of mud crab. PGN, Sp-SPH, PGN/Sp-SPH, PGN/Sp-ALF2, or PGN/BSA was added to the mud crab HLS, respectively. The PO activity of HLS was then determined by using L-dopa as substrate and defined as U/mL. Significant difference in PO activity was marked as stars (paired *t* -test, *P*<0.05). HLS: hemocyte lysate supernatant; PGN: peptidoglycan+HLS; SPH: Sp-SPH+HLS; PGN+SPH: peptidoglycan+ Sp-SPH protein+HLS; PGN+ALF2: peptidoglycan+antilipopolysaccharide factor2+HLS; PGN+BSA:peptidoglycan+bovine serum albumin+HLS. This experiment was repeated three times and the data represented means of triplicates. Bars indicated mean ± S.E. (*n* = 3).

The proPO activating system, present in the hemolymph of crustaceans and other arthropods, is regarded as a crucial component of the immune system and plays a critical role in immune defense against pathogens [29]. To determine whether Sp-SPH protein was involved in the proPO-system activation in the presence of PGN, the PO activity was examined after the addition of PGN together with Sp-SPH to the HLS of mud crab. As indicated in Fig. 5, the PO activity with the addition of Sp-SPH was approximately 1.4–2.5 folds higher than those of controls compared. Significant differences in PO activity were also observed in Sp-SPH supplied sample in comparison with those of the control treatments (paired t-test, *P*<0.05). Besides, we also tested the PO activity in the presence of Sp-SPH protein in crab HLS, with LPS or β-1, 3-glucan as an elicitor of proPO-system activation, but no significant difference was found if LPS or β-1, 3-glucan was present (data not shown). This result obviously indicated that Sp-SPH may function as a co-factor for proPO-system activation triggered by PGN in the mud crab, which was similar to the findings that two crayfish SPH-containing proteins (Pl-SPH1 and Pl-SPH2) are involved in the proPO-system activation when triggered by a Lys-type PGN [30]. Previous studies have reported that clip-domain serine proteinases function as key co-factors for the activation of proPO cascade in arthropods [31]. Meanwhile, the non-catalytic clip domain containing serine proteinase homologues have also been found to act as important co-factors involved in the activation/regulation of the proPO-system in arthropods, including *Anopheles* [32], *Holotrichia diomphalia* [33], *T*. *molitor* [34], *M*. *sexta* [35], [36], *Cotesia rubecula* [37] and *P*. *leniusculus* [30]. With addition of Sp-SPH protein in the present study, the PO activity triggered by PGN, but not LPS or β-1, 3-glucan, was significantly higher than those of controls in mud crab HLS, indicating that Sp-SPH mediated proPO-system activation was in a PGN dependent way in the mud crab. But how this Sp-SPH is recruited for the PGN binding and proPO-system activation still needs further investigations such as determination of the putative proteins complex including the peptidoglycan recognition protein, prophenoloxidase activating factor or phenoloxidase.

### Cumulative mortality of mud crab challenged with bacteria pre-coated with Sp-SPH protein

**Figure 6:**
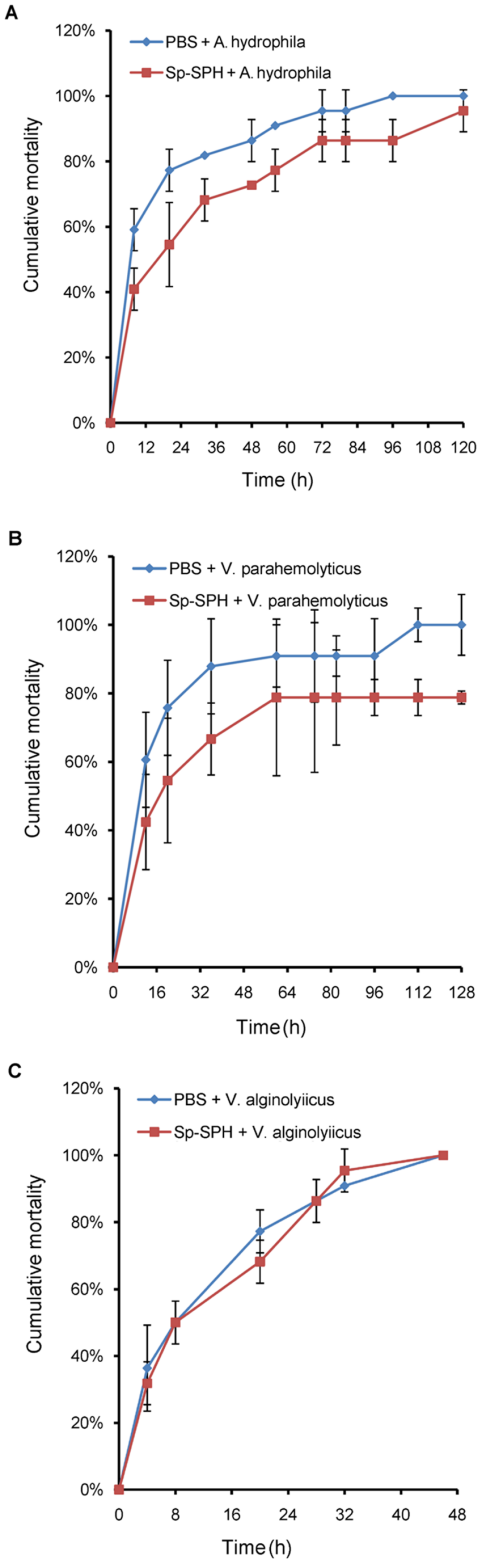
Cumulative mortality of mud crab challenged with bacteria pre-coated with Sp-SPH protein. Crabs were challenged with *A. hydrophila*, *V. parahemolyticus* or *V. alginolyiicus* pre-coated with Sp-SPH protein as described above. Ten animals were used for each group. The mortality was recorded hourly. The cumulative mortality of crabs, in which the bacteria were coated with Sp-SPH protein (square, SPH), was compared with that of animals treated with “PBS-coated” bacteria (diamond, PBS). A: The cumulative mortality caused by *A. hydrophila*; B: The cumulative mortality caused by *V. parahemolyticus*; C: The cumulative mortality caused by *V. alginolyiicus*. This experiment was repeated three times and the data represented means of triplicates. Bars indicated mean ± S.E. (*n* = 3).

We found that Sp-SPH protein displayed different binding affinity to the bacteria tested. According to the hypothesis that the higher PO activity resulted from Sp-SPH activation might lead to higher rate of melanin synthesis and protective efficiency, we performed the cumulative mortality assay of mud crab after challenge with different pathogenic bacteria such as *A. hydrophila* and *V. parahemolyticus*, which were both recognized by the Sp-SPH protein. The cumulative mortality of crab caused by another bacterium, *V. alginolyiicus*, not recognized by Sp-SPH protein under our experimental design, was also determined. As shown in Fig. 6A and Fig. 6B, the cumulative mortality of the crab challenged by *A. hydrophila* or *V. parahemolyticus*, if pre-coated with Sp-SPH protein, was clearly lower than those of animals treated with “PBS-coating bacteria”. Especially, more than 20% of the animals in Sp-SPH protein treated group could survive till seven days post the *V. parahemolyticus* challenge. In case of *V. alginolyiicus* that was not recognized by Sp-SPH protein, both the Sp-SPH protein coated bacterium and non-Sp-SPH protein treated bacterium resulted in similar cumulative mortality, in which all the crabs died within 48 h (Fig. 6C). This finding was in agreement with the studies, when PO activity was increased the host exhibited higher resistance to pathogenic bacterial infection [38], [39]. These data emphasized that Sp-SPH protein may play an important role, probably by bacterial recognition, by enhancing the proPO-system activation for melanin synthesis and promoting cell adhesion activity, in mud crab defense against invading bacteria.

## Conclusion

In summary, our data together clearly suggested that Sp-SPH protein is a multifunctional factor which acts in the recognition of bacteria, activation of the proPO-system and in promoting cell adhesive activity in the mud crab *S. paramamosain*. Further studies are still necessary to elucidate how the Sp-SPH protein distinguishes different bacteria, and possibly interacts with other key molecules such as putative peptidoglycan recognition protein, prophenoloxidase activating factor or prophenoloxidase to enhance the melanin synthesis. These studies will be useful for shedding light on the innate immune defense against pathogenic bacteria in the mud crab, which could be helpful for disease control and selection of fine breeding in the crab farming.

## Supporting Information

Figure S1 **Observation of the cell viability of mud crab hemocyte.** Propidium iodide (PI) is widely used for red-fluorescent nuclear and chromosome counter staining since PI is not permeant to live cells. Hence, PI is also commonly used to detect dead cells in a population. A–C: One hundred microliter of crab hemocyte suspension (1×10^6^ cells/mL), without Sp-SPH protein, was incubated for 1 h in the cell culture plate and stained with PI. D-F: One hundred microliter of crab hemocyte suspension (1×10^6^ cells/mL) containing 4.8 µg Sp-SPH protein was incubated for 1 h in the cell culture plate and strained with PI.
(TIF)

Figure S2 **Determination of mud crab hemocyte viability for cell adhesion assay.** We examined the crab hemocyte viability by using the PI as described in the references [24], [25]. By calculation of the crab hemoctyes, about 96% of crab hemocyte viability was observed via PI staining. No significant difference of the cell viability was observed between Sp-SPH protein treated cells and non-Sp-SPH treated cells.
(TIF)

Figure S3 **Adhesive cells counted under microscope.** The crab hemocytes were prepared as described in the Materials and methods. After washing, the adhesive hemocytes were counted before fixation. According to the 50 µm scale, the cell picture was taken with a 20× objective lens. The hemocytes in the area of 4.256×10^−3^ cm^2^ were counted. By calculation of the crab hemoctyes, there is about 1062 cells in the control sample and approximately 1878 cells in the Sp-SPH protein coating samples. This result indicated that the number of adhesive hemocytes in Sp-SPH protein coating sample (4.4×10^5^ cells/cm^2^) was obviously more than that of the control sample (2.5×10^5^ cells/cm^2^), suggesting a clear cell adhesion activity mediated by Sp-SPH protein. A: Control hemocyte without Sp-SPH protein coating; B: Hemocyte with Sp-SPH protein coating (4.8 µg/well).
(TIF)
