# Supplementary figures and images for: Identification of a Serine Proteinase Homolog (Sp-SPH) Involved in Immune Defense in the Mud Crab Scylla paramamosain
Source: PLoS One. 2013 May 28;8(5):e63787. doi: 10.1371/journal.pone.0063787 (PMC3665817; doi:10.1371/journal.pone.0063787)

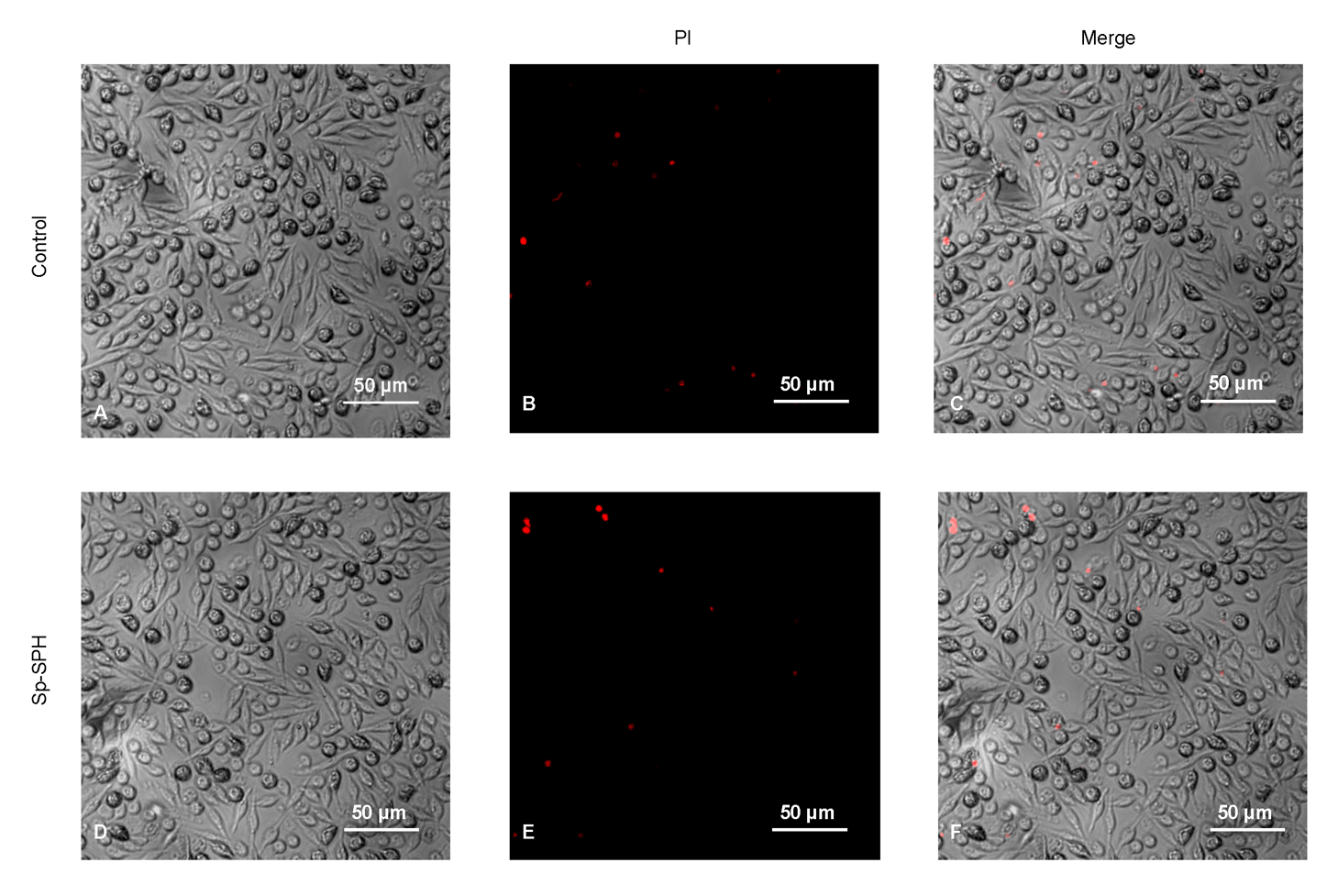

Supplement: Figure S1 — Observation of the cell viability of mud crab hemocyte. Propidium iodide (PI) is widely used for red-fluorescent nuclear and chromosome counter staining since PI is not permeant to live cells. Hence, PI is also commonly used to detect dead cells in a population. A–C: One hundred microliter of crab hemocyte suspension (1×10^6^ cells/mL), without Sp-SPH protein, was incubated for 1 h in the cell culture plate and stained with PI. D-F: One hundred microliter of crab hemocyte suspension (1×10^6^ cells/mL) containing 4.8 µg Sp-SPH protein was incubated for 1 h in the cell culture plate and strained with PI. (TIF) [file pone.0063787.s001.tif]

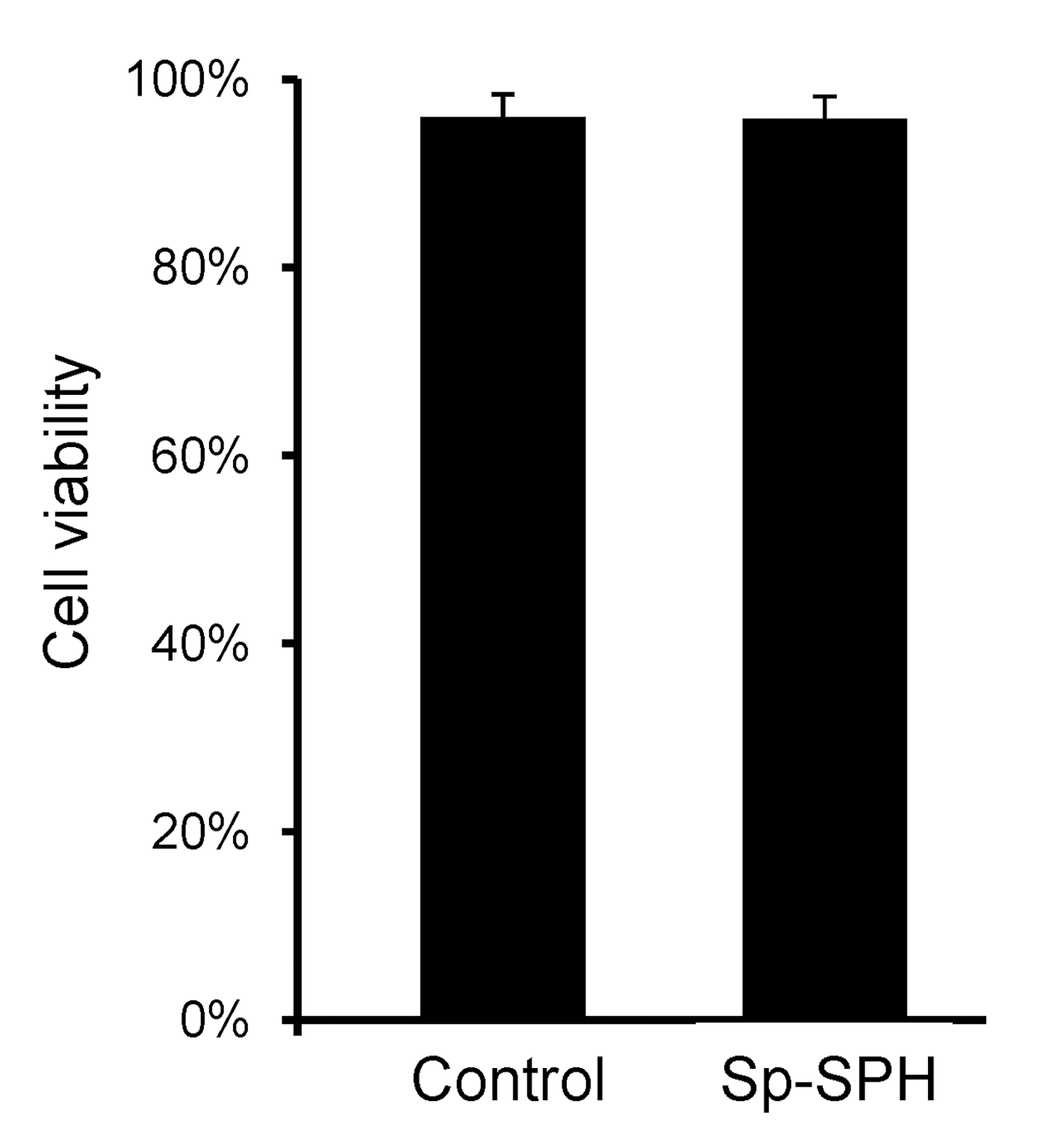

Supplement: Figure S2 — Determination of mud crab hemocyte viability for cell adhesion assay. We examined the crab hemocyte viability by using the PI as described in the references [24], [25]. By calculation of the crab hemoctyes, about 96% of crab hemocyte viability was observed via PI staining. No significant difference of the cell viability was observed between Sp-SPH protein treated cells and non-Sp-SPH treated cells. (TIF) [file pone.0063787.s002.tif]

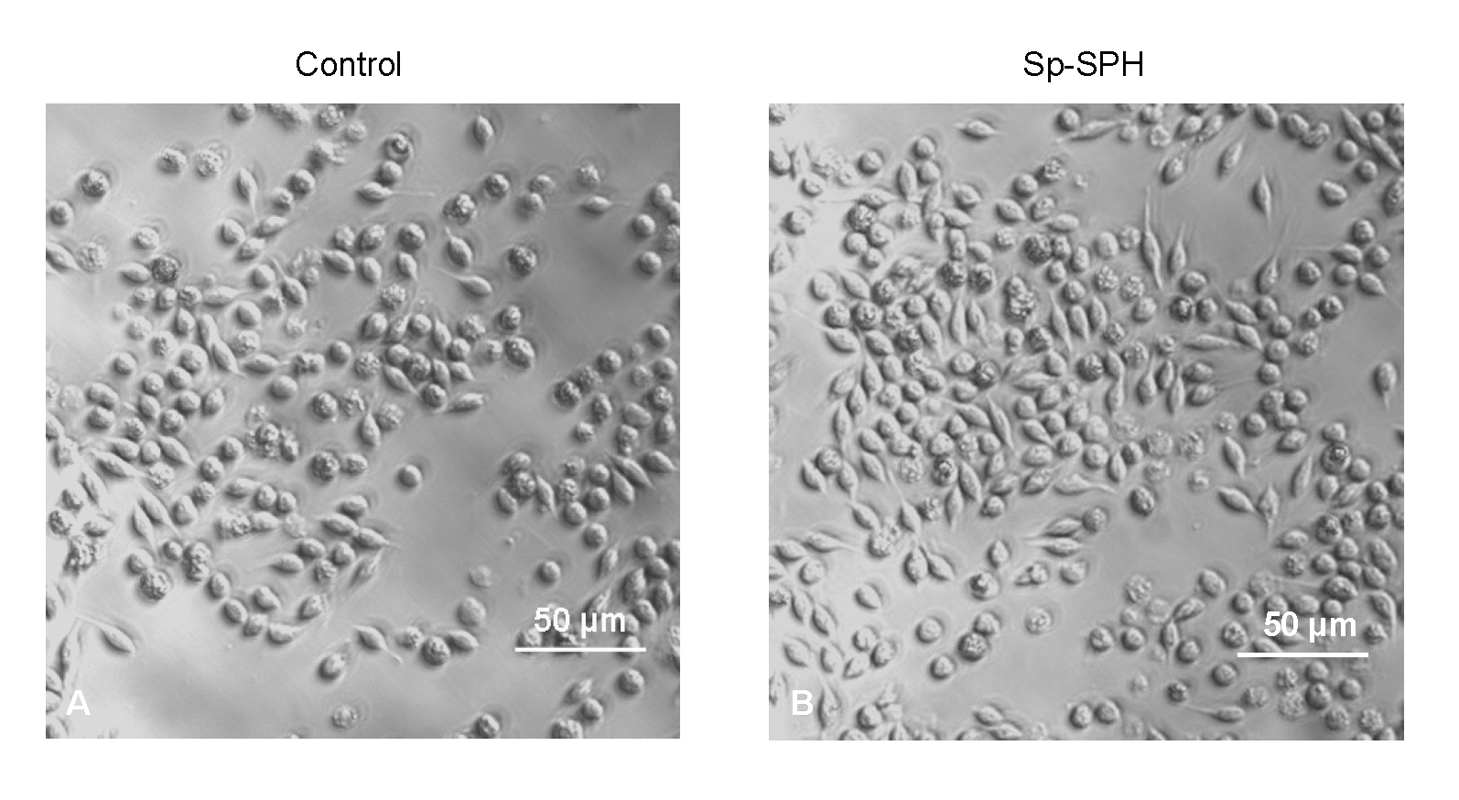

Supplement: Figure S3 — Adhesive cells counted under microscope. The crab hemocytes were prepared as described in the Materials and methods. After washing, the adhesive hemocytes were counted before fixation. According to the 50 µm scale, the cell picture was taken with a 20× objective lens. The hemocytes in the area of 4.256×10^−3^ cm2 were counted. By calculation of the crab hemoctyes, there is about 1062 cells in the control sample and approximately 1878 cells in the Sp-SPH protein coating samples. This result indicated that the number of adhesive hemocytes in Sp-SPH protein coating sample (4.4×10^5^ cells/cm2) was obviously more than that of the control sample (2.5×10^5^ cells/cm2), suggesting a clear cell adhesion activity mediated by Sp-SPH protein. A: Control hemocyte without Sp-SPH protein coating; B: Hemocyte with Sp-SPH protein coating (4.8 µg/well). (TIF) [file pone.0063787.s003.tif]
